# Exploration of Deregulated Long Non-Coding RNAs in Association with Hepatocarcinogenesis and Survival

**DOI:** 10.3390/cancers7030865

**Published:** 2015-09-10

**Authors:** Jing Shen, Abby B. Siegel, Helen Remotti, Qiao Wang, Yueyue Shen, Regina M. Santella

**Affiliations:** 1 Department of Environmental Health Sciences, Mailman School of Public Health, Columbia University Medical Center, New York, NY 10032, USA; E-Mails: qw6@cumc.columbia.edu (Q.W.); Yueyue.Shen@uvm.edu (Y.S.); rps1@cumc.columbia.edu (R.M.S.); 2 Herbert Irving Comprehensive Cancer Center, Columbia University Medical Center, New York, NY 10032, USA; 3 Department of Medicine, Columbia University Medical Center, New York, NY 10032, USA; E-Mail: aas54@cumc.columbia.edu; 4 Department of Pathology and Cell Biology, Columbia University Medical Center, New York, NY 10032, USA; E-Mail: her2007@cumc.columbia.edu

**Keywords:** long non-coding RNAs, deregulation, HCC, HBV, survival

## Abstract

Long non-coding RNAs (lncRNAs) are larger than 200 nucleotides in length and pervasively expressed across the genome. An increasing number of studies indicate that lncRNA transcripts play integral regulatory roles in cellular growth, division, differentiation and apoptosis. Deregulated lncRNAs have been observed in a variety of human cancers, including hepatocellular carcinoma (HCC). We determined the expression profiles of 90 lncRNAs for 65 paired HCC tumor and adjacent non-tumor tissues, and 55 lncRNAs were expressed in over 90% of samples. Eight lncRNAs were significantly down-regulated in HCC tumor compared to non-tumor tissues (*p* < 0.05), but no lncRNA achieved statistical significance after Bonferroni correction for multiple comparisons. Within tumor tissues, carrying more aberrant lncRNAs (6–7) was associated with a borderline significant reduction in survival (HR = 8.5, 95% CI: 1.0–72.5). The predictive accuracy depicted by the AUC was 0.93 for HCC survival when using seven deregulated lncRNAs (likelihood ratio test *p* = 0.001), which was similar to that combining the seven lncRNAs with tumor size and treatment (AUC = 0.96, sensitivity = 87%, specificity = 87%). These data suggest the potential association of deregulated lncRNAs with hepatocarcinogenesis and HCC survival.

## 1. Introduction

The incidence of hepatocellular carcinoma (HCC) in the United States has tripled over the past 30 years [1,2,3]. HCC has an extremely poor prognosis if not diagnosed and treated at an early stage. The average 5-year survival rate is less than 12% [4], and only 3% in advanced disease. Therefore, early detection of HCC is crucial for successful curative treatment. The aberrant expression of protein-coding genes play an important role in hepatocarcinogenesis, but these genes only account for 1%–2% of transcribed RNAs [5,6,7].

A recent human transcriptome study revealed that a large number of non-coding RNAs (ncRNAs), including long ncRNA (lncRNA), are abundant in human tissues and crucial regulators of cellular transcription and translation [5,8,9]. LncRNA, larger than 200 nucleotides, are transcribed from intergenic and intragenic regions and constitute a broad class of cellular transcripts [10,11,12,13,14]. They play a critical role in the regulation of gene expression through chromatin remodeling, RNA maturation (splicing, editing), transport and protein synthesis [15]. Initial evidence suggests that lncRNAs have essential roles in tumorigenesis [16] and tumor progression [17,18]. Recent studies revealed that deregulated lncRNAs are found in a number of human cancers [19], including HCC [20,21,22,23,24,25,26,27,28,29,30]. Those aberrantly expressed in HCC tumor compared with non-tumor tissue include *HOTAIR* (HOX antisense intergenic RNA), *HULC* (highly upregulated in liver cancer), *MALAT1* (metastasis‑associated lung adenocarcinoma transcript 1), *MEG3* (maternally expressed gene 3), *MVIH* (microvascular invasion in HCC), and *UCA1* (urothelial carcinoma-associated 1) [20,21,22,23,24,25,26,27,28,29,30]. HCC recurrence, metastasis and prognosis are also predicted by altered lncRNAs including *GAS5* (growth arrest-specific transcript 5), *HEIH* (high expression in HCC), *HOTAIR*, *HOTTIP* (HOXA transcript at the distal tip), *MALAT1* and *UCA1* [23,28,30,31,32,33]. However, due to their small sample sizes, these prior studies had limited statistical power to identify reliable lncRNA biomarkers associated with hepatocarcinogenesis and prognosis. The potential impacts of HCC etiologies (hepatitis B virus (HBV) and hepatitis C virus (HCV) infection) for lncRNAs expression are also unclear. 

Using available paired HCC tumor and adjacent non-tumor tissues collected by the Center for Liver Disease and Transplantation and the Herbert Irving Comprehensive Cancer Center (HICCC), Columbia University Medical Center (CUMC), we determined the expression profiles of 90 cancer related lncRNAs, and explored their potential association with hepatocarcinogenesis, hepatitis virus infection or HCC survival.

## 2. Materials and Methods

### 2.1. Patients and Tissue Samples

This study was approved by the Institutional Review Board of CUMC. Sixty-six frozen tumor and paired adjacent non-tumor tissues were obtained from HCC patients who underwent either surgical resection or liver transplant at CUMC. Histological evaluation was performed in the Molecular Pathology Shared Resource of the HICCC by the study pathologist (H.R.). Tumor samples were macrodissected to assess presence and percent of tumor and ensure >80% purity of tumor. Tumor stage was determined according to the American Joint Committee on Cancer (AJCC) criteria [34]. Separate blocks of non-tumor liver tissues were evaluated with respect to presence (Batts-Ludwig stage of 4) or absence of cirrhosis (Batts-Ludwig stage < 4). Information on viral infection (HBV, HCV) and clinicopathological features including α-fetoprotein levels, tumor size, tumor number, tumor differentiation, vascular invasion, and capsular infiltration were obtained from the medical records.

### 2.2. RNA Extraction and lncRNA Measurement

Total RNA was extracted from HCC tumor and adjacent non-tumor tissues by RNeasy Microarray Tissue Mini Kits (Qiagen, Frederick, MA, USA) according to the manufacturer’s protocol. RNA quantification and quality were evaluated with an Agilent 2100 Bioanalyzer. The Lnc Profiler™ qPCR Array (System Biosciences (SBI), Mountain View, CA, USA) was used to measure the expression of 90 lncRNAs that were chosen using the following criteria: (1) LncRNA sequence was well curated and accepted; (2) in one or more publications lncRNA was implication in human cancer and stem cells; (3) Primer sets and sequences were available in prior publications; (4) Primer sets passed internal SBI quality control for specificity performance. Five housekeeping genes (*18S rRNA*, *RNU43*, *GAPDH*, *LAMIN A/C*, and *U6*) and one negative control were used as reference controls to adjust the expression of candidate lncRNAs (Supplementary Table S1). One sample failed array detection, and was omitted from final data analysis. Briefly, 1.2 µg isolated RNA (5 µL) was mixed with reagents (PolyA Buffer, MnCl_2_, ATP and PolyA Polymerase) to polyadenylate all lncRNAs. Then the oligo dT adaptor and random primers were added and samples incubated at 42 °C for 60 min., and heated at 95 °C for 10 min. to complete cDNA conversion to enhance qPCR assay performance. Finally, the expression profiles for lncRNAs were determined by SYBR Green based qPCR run in 96-well plates on an Applied Biosystems 7500 Real-time PCR System. The cycle of threshold (Ct) was determined for each lncRNA, and the raw Ct values were normalized by the geometric mean of the 5 housekeeping genes to indicate lncRNA expression level. The relative amount of each lncRNA was described as fold-change between tumor and non-tumor tissues using the equation of 2^−∆∆Ct^ [35]. A representative result of lncRNAs fold-change is shown in Supplementary Figure S1.

### 2.3. Statistical Analysis

The expression levels of lncRNAs were displayed as log_2_ transferred geometric mean. Paired *t*-test was used to analyze the expression difference between tumor and adjacent non-tumor tissues. Two-sample *t* test was used to determine the expression differences by HBV and HCV status within non-tumor tissues. Kaplan-Meier survival analysis and log rank test were used to assess differences of survival months by aberrant lncRNAs status (categorized by the median in survival cases). Cox proportional hazard models were conducted to determine the impact of lncRNAs and clinicopathologic parameters on overall survival (defined as the time between surgical resection or liver transplant and death from any cause or last follow-up). Age and survival months were treated as continuous variables, while type of surgery (resection *vs.* transplant), gender, ethnicity, virus infection status, cirrhosis, tumor size (≥4 cm *vs.* < 4 cm), and tumor grade (IV *vs.* III *vs.* I–II) were treated as categorical variables. Logistic regression was used to construct receiver operating characteristic (ROC) curves for each lncRNA and clinical factor that may potentially predict HCC survival. Finally, 7 lncRNAs (as continuous variables), tumor size and type of surgery (as categorical variables) were chosen using a stepwise model selection method to construct an optimal model. The maximum sensitivity, specificity and the area under the curve (AUC) were estimated using 0.5 probability of death as the cutoff point [36]. All statistical analyses were performed using Statistical Analysis System 9.0 (SAS Institute, Cary, NC, USA).

## 3. Results

### 3.1. Clinical and Pathological Characteristics

Table 1 shows the clinical and pathological characteristics of the 65 HCC patients. The average age of cancer diagnosis is 59.5 years, and over half are older than 60 years (57%). Most patients are male (75%) and Caucasian (51%). The HBV and HCV infection frequencies are, respectively 20% and 29%, similar to that of being both virus negative (28%). Among all patients, 72% have pathologically defined cirrhosis and 60% have grade III or IV tumors.

**Table 1:** Clinical and pathological characteristics.

Variables	No. of Cases (%)
Age at diagnosis (yrs), Mean ± SD	59.5 ± 14.7
Age group	
<60 years	28 (43)
≥60 years	37 (57)
Gender	
Male	49 (75)
Female	16 (25)
Ethnicity	
Caucasian	33 (51)
African-American	6 (9)
Hispanic	6 (9)
Asian	14 (22)
Unknown/Other	6 (9)
Viral infection	
HBV (−), HCV (−)	18 (28)
HBV (−), HCV (+)	19 (29)
HBV (+), HCV (−)	13 (20)
HBV (+), HCV (+)	4 (6)
Missing	11 (17)
Cigarette smoking	
No	26 (40)
Yes	35 (54)
Missing	4 (6)
Alcohol drinking	
No	27 (42)
Yes	35 (54)
Missing	3 (4)
Cirrhosis	
No	17 (26)
Yes	47 (72)
Missing	1 (2)
Tumor size (cm), Mean ± SD	6.1 (4.8)
Tumor grade *	
I–II	23 (35)
III	21 (32)
IV	18 (28)
Missing	3 (5)

* Edmondson and Steiner grade.

### 3.2. Differentially Expressed lncRNAs in HCC Tissues

A total of 90 lncRNAs were measured in the current study, and a Volcano Plot used to show expression differences between tumor and non-tumor tissues (Supplementary Figure S2). Among them, 55 lncRNAs expressed in over 90% of samples were analyzed. Eight (*lincRNA-VLDLR*, *MEG9*, *H19 antisense*, *ncR-uPAR*, *NEAT1 (family)*, *LUST*, *UM9-5*, and *HOTAIR*) were significantly down-regulated in HCC tumor compared to non-tumor tissues at a significance level of *p* < 0.05 (Table 2). The fold changes ranged from −2.1 to −2.8. However, after Bonferroni correction for multiple comparisons, no lncRNA marker achieved statistical significance with false discovery rates (FDR) ranging from 0.19 to 0.29. Previously, four lncRNAs (*H19*, *HOTAIR*, *HULC* and *MALAT1*) were reported to be associated with HCC, but only *HOTAIR* was borderline significance (Table 2) in the current study. These results may be due to the heterogeneity of HCC tumors with various etiologies.

**Table 2:** Deregulated lncRNAs expression (Log_2_ of geometric mean) in HCC tumor compared with non-tumor tissues (*N* = 65 pairs).

lncRNAs	Tumor	Non-Tumor	Fold-Change	Unadjusted *p*-Value	FDR
*lincRNA-VLDLR*	−4.6	−3.4	−2.4	0.005	0.188
*MEG9*	−5.6	−4.2	−2.8	0.010	0.188
*H19 antisense*	−1.3	0.2	−2.7	0.010	0.188
*ncR-uPAR*	−1.3	−0.03	−2.6	0.018	0.188
*NEAT1 (family)*	−2.4	−1.3	−2.2	0.021	0.188
*LUST*	−0.5	0.6	−2.1	0.037	0.287
*UM9-5*	−8.7	−7.6	−2.5	0.042	0.287
*HOTAIR*	−8.8	−7.1	−2.6	0.048	0.287

### 3.3. Deregulated lncRNAs in HBV- or HCV-Related HCC

Categorizing tissues by viral status, we separately analyzed the expression patterns of lncRNAs in HBV or HCV-related HCC. Seven lncRNAs were significantly repressed in HBV positive tumors compared with paired non-tumor tissues (Table 3). including two markers first identified in HCC—*Kcnq1ot1* (KCNQ1 overlapping transcript 1), a well-characterized tumor suppressor [37] and *NRON* [noncoding repressor of NFAT (nuclear factor of activated T-cells)] [38] to repress NFAT functions involved in carcinogenesis, cancer cell proliferation and metastasis [39,40]. The fold changes of *Kcnq1ot1* and *NRON* ranged from −5.3 to −9.1. Similarly, no significant lncRNA was found between HBV-positive tumor and adjacent non-tumor tissues after Bonferroni correction for multiple comparisons. No significant lncRNA distinguished HCV positive tumors (*N* = 19), or viral negative tumors (*N* = 18) from paired non-tumor tissues.

**Table 3:** Deregulated lncRNAs expression (Log_2_ of geometric mean) in HBV-related HCC tumor compared with non-tumor tissues (*N* = 13 pairs).

lncRNAs	Tumor	Non-Tumor	Fold-Change	Unadjusted *p*-value	FDR
*Kcnq1ot1*	−8.9	−5.2	−9.1	0.005	0.215
*TncRNA*	−5.5	−4.0	−2.9	0.009	0.215
*lincRNA-VLDLR*	−3.8	−1.8	−4.2	0.014	0.215
*Zfhx2as*	−2.0	0.8	−6.7	0.019	0.215
*NRON*	−6.1	−3.7	−5.3	0.020	0.215
*HOTTIP*	−9.8	−7.8	−4.2	0.025	0.231
*lincRNA-RoR*	−9.5	−7.7	−3.4	0.046	0.273

We separately compared lncRNAs patterns within tumor and non-tumor tissues by viral status. No lncRNAs were significantly differentially expressed by viral status within tumor tissue comparisons. Only *Kcnq1ot1* and *NRON* were significantly up-regulated in HBV positive compared with viral negative non-tumor tissues (Table 4). The fold changes were from 5.4 to 12.6. No significant lncRNA was obtained after adjusting for multiple comparisons.

**Table 4:** Deregulated lncRNAs (Log_2_ of geometric mean) significantly associated with HBV infection in HCC non-tumor tissues.

LncRNAs	Viral Negative (*N* = 18)	HBV Positive (*N* = 13)	Fold-Change	Unadjusted *p*-Value	FDR
*Kcnq1ot1*	−8.9	−5.2	12.6	0.004	0.193
*NRON*	−6.2	−3.7	5.4	0.042	0.947

### 3.4. Aberrant Expression of lncRNAs in Tumor Tissue and Prediction for HCC Survival

**Figure 1:**
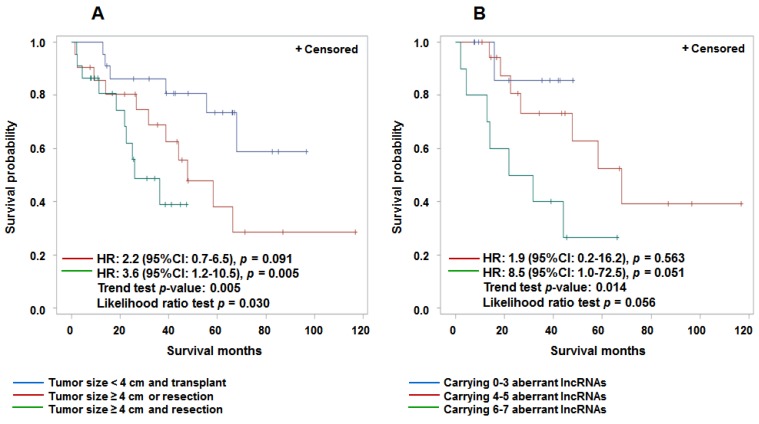
Kaplan-Meier survival curves to assess aberrantly expressed lncRNAs, tumor size and treatment in prediction of HCC survival. (**A**) shows that large tumor size (≥4 cm) and resection treatment were significantly associated with reduced HCC survival compared with small tumor size (<4 cm) and liver transplant treatment; (**B**) shows that carrying more aberrantly expressed lncRNAs is associated with a reduction in survival compared with carrying less aberrant lncRNAs.

We examined the potential role of aberrantly expressed lncRNAs in tumor tissue and prediction of HCC survival and mortality. Seven aberrantly expressed lncRNAs were observed including two up-regulated (*Kcnq1ot1*, *PRINS*) and five repressed (*21A*, *SNHG4*, *BACE1AS* (family), *UCA1*, *Tmevpg1*) lncRNAs in HCC cases with poor survival (Supplementary Table S2). The fold changes ranged from −4.7 to 3.7. Individual lncRNA can predict a short HCC survival time with hazard ratios (HR) ranging from 1.7 to 3.0 after adjustment for age and gender (Supplementary Figure S3). These values are similar to those for large tumor size (HR = 1.8, 95% CI: 0.8–4.1) and treatment by resection alone (HR = 5.6, 95% CI: 2.1–15.0)—two known clinical predictors of HCC prognosis. However, only up-regulated *Kcnq1ot1* and treatment of resection achieved statistical significance (*p* < 0.05). Patients with both larger tumor size and a resection had significantly reduced survival (HR = 3.6, 95% CI: 1.2–10.5) compared with those having small tumors or liver transplant (Figure 1A). Carrying more aberrant lncRNA markers (6–7) also showed a borderline significant reduction in survival (HR = 8.5, 95% CI: 1.0–72.5) compared with carrying fewer (0–3) markers (Figure 1B), indicating the potential role of a panel of lncRNAs, not individual lncRNA, in prediction of HCC prognosis.

**Figure 2:**
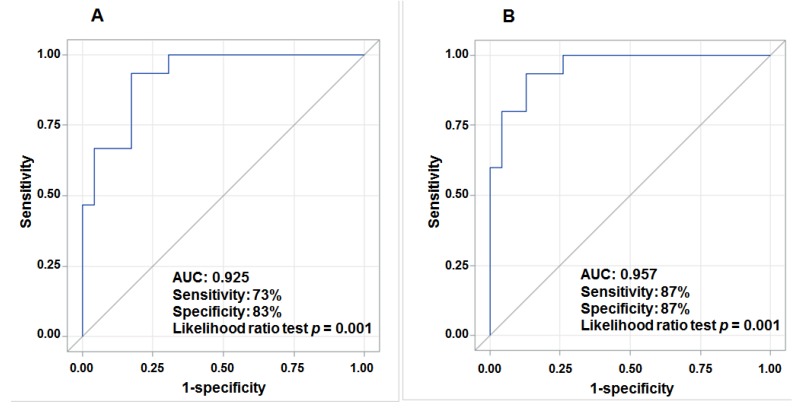
ROC curves of deregulated lncRNAs, tumor size and type of surgery for prediction of HCC survival. (**A**) shows an AUC of 0.93, a sensitivity of 73%, and a specificity of 83% for the combination of 7 aberrant lncRNAs in tumor tissue in prediction of HCC survival adjusted for age and gender; (**B**) displays an AUC of 0.96 when combining 7 aberrant lncRNAs, tumor size and type of surgery in prediction of HCC survival adjusted for age and gender.

The predictive accuracy depicted by the AUC for individual lncRNA ranged from 0.62 to 0.71 (*p* < 0.20), similar to that for large tumor size (0.63) and resection treatment (0.64) adjusted for age and gender (Supplementary Figure S4). A combination of deregulated *Kcnq1ot1*, *21A*, *SNHG4*, *BACE1AS* (family), *PRINS*, *UCA1* and *Tmevpg1* significantly predicted (likelihood ratio test *p* = 0.001) HCC survival with an AUC of 0.93, 73% sensitivity and 83% specificity (Figure 2A) after adjusting for age and gender. When including seven lncRNAs (continuous variables), tumor size, type of surgery (categorical variables) and covariates (age and gender) in a multivariate logistic regression model, we obtained an optimal HCC survival model with an AUC of 0.96 (87% sensitivity and 87% specificity) (Figure 2B).

## 4. Discussion

We examined 90 lncRNAs in 65 HCC tissues, and identified a panel of lncRNAs repressed in HCC (Table 2), or associated with HBV-infected HCC (Table 3). However, none achieved statistical significance after Bonferroni correction for multiple comparisons indicating the biological role of these lncRNAs in hepatocarcinogenesis needs further clarification.

Four identified lncRNAs have limited data to indicate their biological role in tumorigenesis. *lincRNA-VLDLR* (very low density lipoprotein receptor) belongs to the low density lipoprotein receptor family that has multiple functions in binding numerous ligands, and regulating cellular signaling. *lincRNA-VLDLR* has been detected in HCC tissues and hepatoma cell lines [41], but is repressed in HCC tumor tissue in the current study. Three biological mechanisms may be involved in its deregulation: as a direct target of miR-135a-5p [42]; hypermethylation of the gene (9p24.2) promoter [43] and homozygous loss of the gene observed in genome-wide screening for copy-number alterations in cancer cell lines, albeit infrequently [43]. *LUST* (LUCA-15-Specific Transcript) is mapped to the antisense strand of gene *RBM5* (3p21.3) that functions as a putative tumor suppressor [44]. Ectopic overexpression of *LUST* coincides with elevated expression of full-length *RBM5*, and reduced expression of the truncated, cytotoxic *RBM5*, which inhibits cellular proliferation and enhances apoptosis [45]. The KCNQ1 cluster (11p15) contains approximately 10 paternally imprinted genes, whose expression is regulated by a tumor suppressor *Kcnq1ot1* [37], also known as *LIT1*. Loss of expression of *Kcnq1ot1* in colorectal cancer [46] and repression in skin cancer [47] is mainly controlled by epigenetic modifications, *i.e.*, aberrant DNA methylation, interaction with DNA (cytosine-5-)-methyltransferase 1 (*DNMT1*), enrichment of H3 lysine 9 dimethylation (H3K9me2) and reduction of H3 lysine 4 (H3K4) demethylation [46,47,48]. A novel short tandem repeat (STR) polymorphism in *Kcnq1ot1* was significantly associated with higher expression of Kcnq1ot1 (21–33 folds) and decreased risk of HCC (OR = 0.38, 95% CI: 0.21–0.69) [49]. Although no direct evidence to indicate the anti-cancer role of *NRON*, its repression for NFAT that plays pivotal role in tumorigenesis, cell proliferation, migratory, invasive and drug resistance, suggests a potential tumor suppressive function [39,40]. However, previous studies are not always consistent. The activation of estrogen signaling in breast cancer cells appeared to enhance expression of *Kcnq1ot1* and repression of *CDKN1C* (cyclin-dependent kinase inhibitor 1C) that is concomitant with loss of *Kcnq1ot1* methylation in its promoter CpG island [50]. Similar inverse correlations between *Kcnq1ot1* and *CDKN1C* expression were observed in three hepatoma cell lines [49], indicating a diverse and complex bidirectional regulatory role for lncRNAs in tumorigenesis. This is consistent with our discrepant observations that repressed *Kcnq1ot1* and *NRON* were observed in HBV-related HCC tumor compared with adjacent non-tumor tissues (Table 3), while up-regulated *Kcnq1ot1* and *NRON* were found in HBV-related HCC compared with viral negative non-tumor tissues (Table 4). One explanation is that, when HBV infects liver tissue, the expression of *Kcnq1ot1* (as a tumor suppressor) is activated in order to preclude the carcinogenic effect of HBV infection, or try to compensate for its partially disrupted tumor suppressive function. A similar mechanism may be also applied to *NRON* regulation, which is supported by a recent finding that *NRON* expression can modulate HIV-1 replication, and knockdown of NRON enhances the replication of HIV-1 through increased activity of NFAT [51]. It is biologically plausible given the features of lncRNAs that regulate protein-coding gene expression at both post-transcriptional and transcriptional levels [52]. For transcriptional regulation, lncRNAs can recruit chromatin-modifying enzymes to positively or negatively control a protein-coding gene’s expression, either *in cis* (near the site of lncRNA) or *in trans* (the involved genes are distant) [53]. Other potential mechanisms involved in these discrepant effects of deregulated lncRNAs need further exploration in hepatocarcinogenesis. 

We found that seven lncRNAs in tumor tissues has pronounced predictive capability for HCC survival (HRs from 1.7 to 3.0), but only one achieves statistically significant level (Supplementary Figure S3). Carrying 6–7 aberrantly expressed lncRNAs was associated with a borderline significant reduction in survival (HR = 8.5, 95% CI: 1.0–72.5) compared with carrying fewer aberrant markers (Figure 1). This data suggests that a panel of deregulated lncRNAs may serve as a marker to predict HCC survival. Several previous studies have observed that a few different lncRNAs are independent predictors of HCC prognosis [23,28,30,31,32,33]. Studies in liver transplant patients found that overexpression of *HOTAIR* and *MALAT1* were independent predictors for HCC recurrence. The HRs were, respectively 3.6 (95% CI: 1.7–7.6) and 3.3 (95% CI: 1.5–7.1) [23,28]. Overexpressed *HOTTIP* was significantly associated with increased metastasis and decreased overall survival for HCC patients with mixed etiologies [31]. A high level of *HEIH* has been found significantly associated with HBV-related HCC recurrence (HR = 2.1; 95% CI: 1.2–3.7) [33], while another study found HCC patients with lower levels of *GAS5* in tumor tissue had a worse overall survival than patients with higher expression (HR = 2.4; 95% CI: 1.6–4.1) [32]. Upregulated *UCA1* was significantly correlated with advanced HCC TNM stage, metastasis and poor 5-year survival [30]. However, these studies did not adjust for patients’ demographic characteristics (age, gender) and clinical pathological factors (tumor size, stage, grade, and treatment status) that are associated with HCC survival. It is unable to completely exclude the potential bias of those factors on lncRNAs expression and HCC survival. 

Most studies using individual lncRNAs as a predictor for cancer survival had much poorer accuracy than those using a panel of lncRNAs. For the first time, we used a panel of seven deregulated lncRNAs to obtain a predictive accuracy (AUC) of 0.93 (Figure 2) in prediction of HCC survival, which is similar to the AUC (0.96) when combing the seven lncRNAs with tumor size and type of surgery (sensitivity = 87%, specificity = 87%). Except for *UCA1*, the other lncRNAs (*Kcnq1ot1*, *21A*, *SNHG4*, *BACE1AS* (family), *PRINS* and *Tmevpg1*) identified in this panel have not been previously reported to be associated with HCC survival. Accumulating evidence from survival studies in other tumors and the analysis of relevant biological pathways or genes support their potential role in prediction of HCC prognosis. Upregulated *UCA1* in HCC can promote progression through a novel UCA1-miR-216b-FGFR1-ERK signaling pathway [30]. *UCA1* has also been found to enhance bladder cancer cell proliferation and metastasis by disrupting the PI3K/Wnt signaling pathway [54,55]. A low expression level of *PRINS* (psoriasis susceptibility-related RNA gene induced by stress) was found to be associated with adrenocortical carcinoma (ACC) recurrence and distant metastatic disease [56] because of its regulation of *G1P3*, an anti-apoptotic gene [57]. Aberrant expression of *UCA1* (urothelial carcinoma-associated 1) and three other lncRNAs were found in the co-expression network with 26 mRNAs involved in the progression of gastric cancer [58]. *BACE1AS* (antisense transcript for β-secretase-1) was downregulated in 5-fluorouracil (5-FU) resistant cells derived from the human colon cancer [59] that may impact the efficacy of treatment and lead to poor prognosis. However, these data are far from sufficient to clearly characterize the functional lncRNAs involved in HCC progression.

Several previous studies have identified panels of deregulated lncRNAs in association with HCC tumors, hepatitis infection, as well as survival, but the results vary greatly. These differences may be due to the heterogeneity of liver tissues with various HCC etiologies (age and gender, HBV, HCV, alcoholic or steatohepatitis) and pathological characteristics (tumor size, stage, cirrhosis, inflammation activities, treatment status, *etc.*). Use of different molecular techniques to measure lncRNAs or selection of different samples (adjacent non-tumor tissue, cirrhotic tissue or normal liver tissues) as comparison tissues may also lead to inconsistent results. The types and numbers of endogenous controls used to normalize lncRNAs expression levels vary by study and may also cause the discrepancy. Therefore, well-designed studies are needed to better elucidate liver specific lncRNA alterations associated with hepatocarcinogenesis, viral etiologies or HCC prognosis. 

One limitation of the current study is lack of gene expression profiles that enable construction of lncRNA-mRNA co-expression networks to further understand relevant biological functions of interesting lncRNAs. When categorized by HCC etiologies or survival status, the subgroups’ sample sizes are small. Although several panels of lncRNAs were aberrantly expressed in either overall HCC or HBV-related HCC or poor survival HCC, no lncRNA achieved statistical significance after Bonferroni correction for multiple comparisons. Therefore, our findings should be interpreted with caution. Further large studies using homogenous HCC etiologies are needed to draw firm conclusions.

## 5. Conclusions

We identified a panel of eight lncRNAs associated with HCC occurrence, seven lncRNAs repressed in HBV-related HCC, and seven lncRNAs aberrantly expressed in HCC patients with poor survival. A combination of the seven lncRNAs and large tumor size and resection treatment can accurately predict HCC survival with an AUC of 0.96 and a sensitivity/specificity of 87%. These data suggest that a panel of lncRNAs may serve as potential markers for HCC early diagnosis and prediction of survival. However, large studies are needed to validate these findings due to the limitations mention above. 

## Supplementary Files

Supplementary File 1
