# Supplementary material for: Exploration of Deregulated Long Non-Coding RNAs in Association with Hepatocarcinogenesis and Survival
Source: Cancers (Basel). 2015 Sep 10;7(3):1847–62. doi: 10.3390/cancers7030865 (PMC4586798; doi:10.3390/cancers7030865)
Supplement: Supplementary File 1 [file cancers-07-00865-s001.pdf]

Supplementary Materials

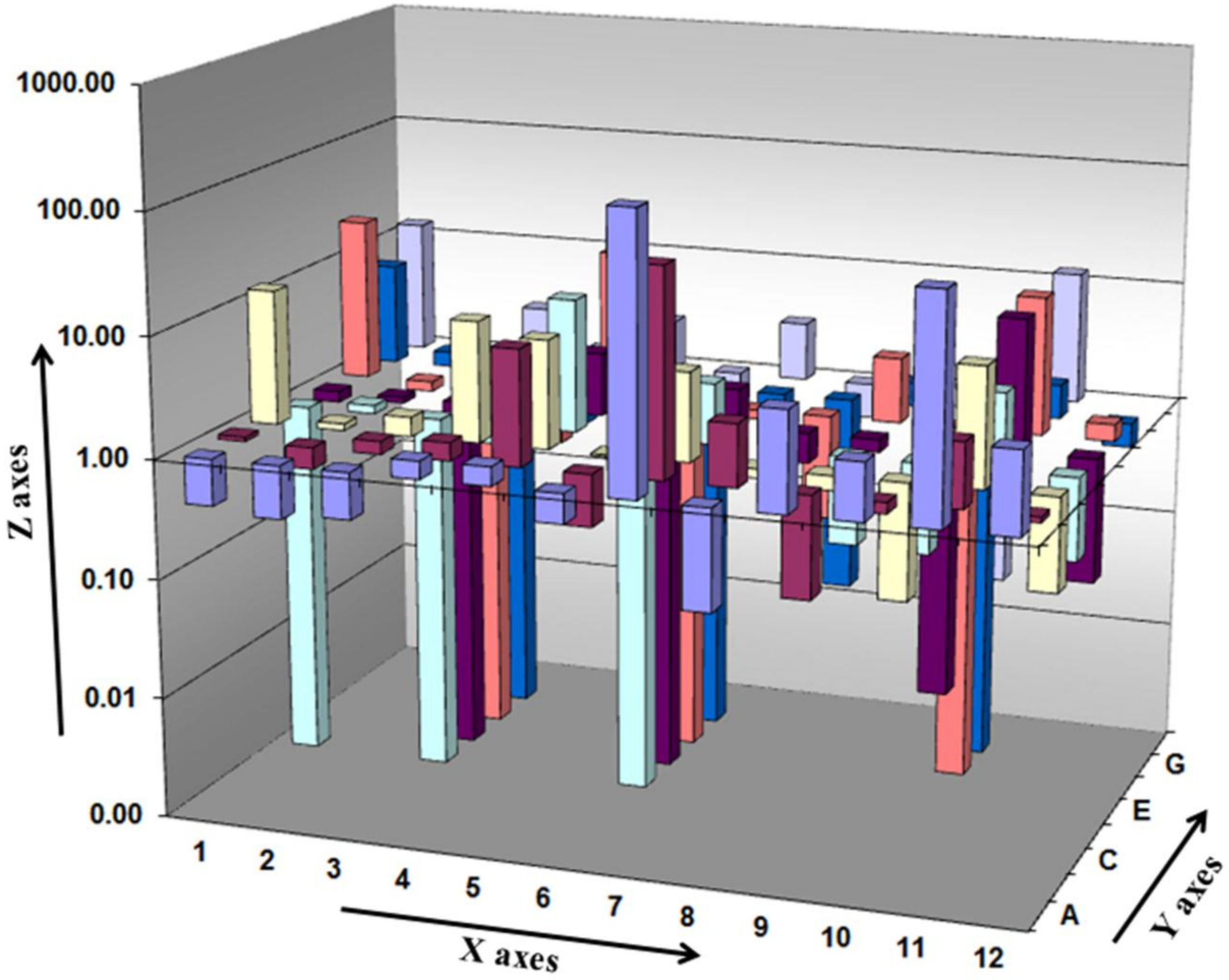

Figure S1. A representative example of the fold changes in expression of lncRNAs between tumor (ID: 6236) and adjacent non-tumor tissues (ID: 6235). X axis (1–12) indicates the column location of specific lncRNA. Y axis (A–H) indicates the line location of specific lncRNA. Z axis (0–1000) indicates the fold change of lncRNA between tumor and paired non-tumor tissues. There is a wide range in variations of expression between tumor and non-tumor tissues, indicating the potential usefulness of aberrant lncRNAs as tumorigenic biomarker.

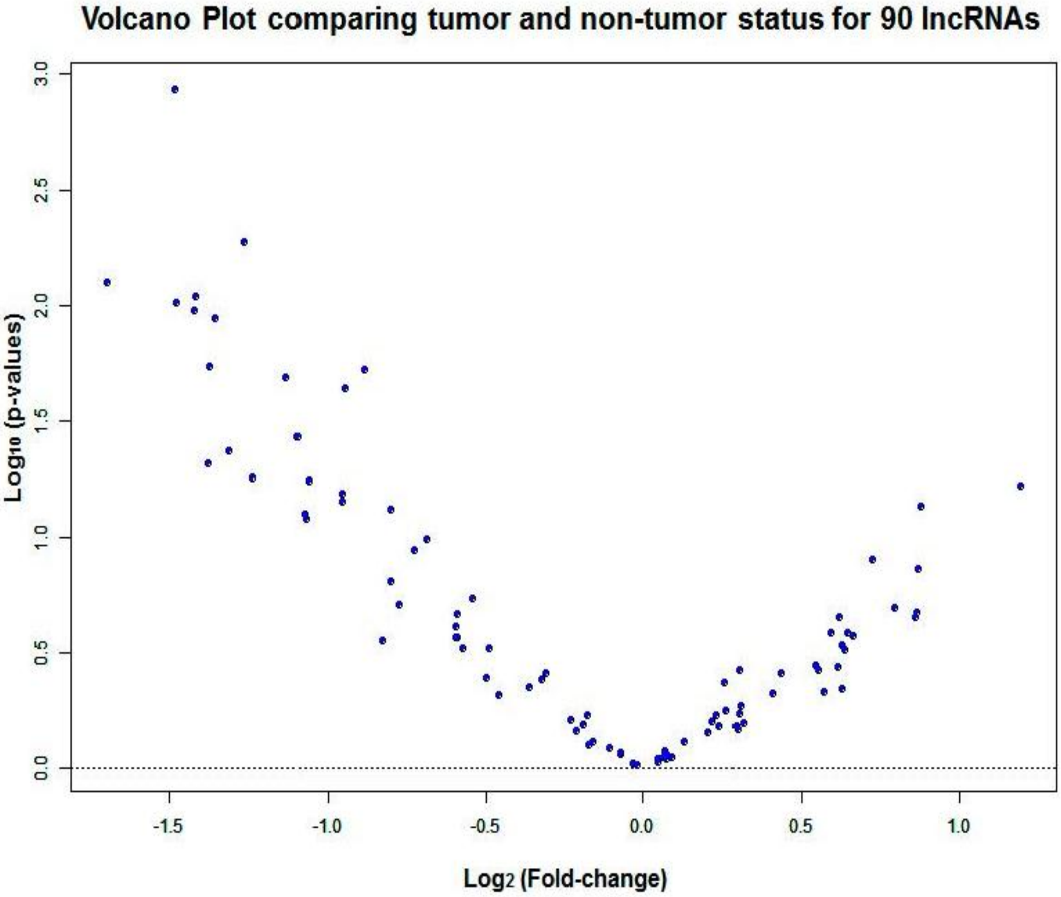

Figure S2. Volcano Plot comparing tumor and non-tumor status for 90 lncRNAs. Over 60% (55/90) of the lncRNAs were detectable in over 90% of liver tissues, suggesting the abundance of lncRNAs expression. Many lncRNAs were down-regulated in tumor tissue, while a few lncRNAs were up-regulated in tumor tissue.

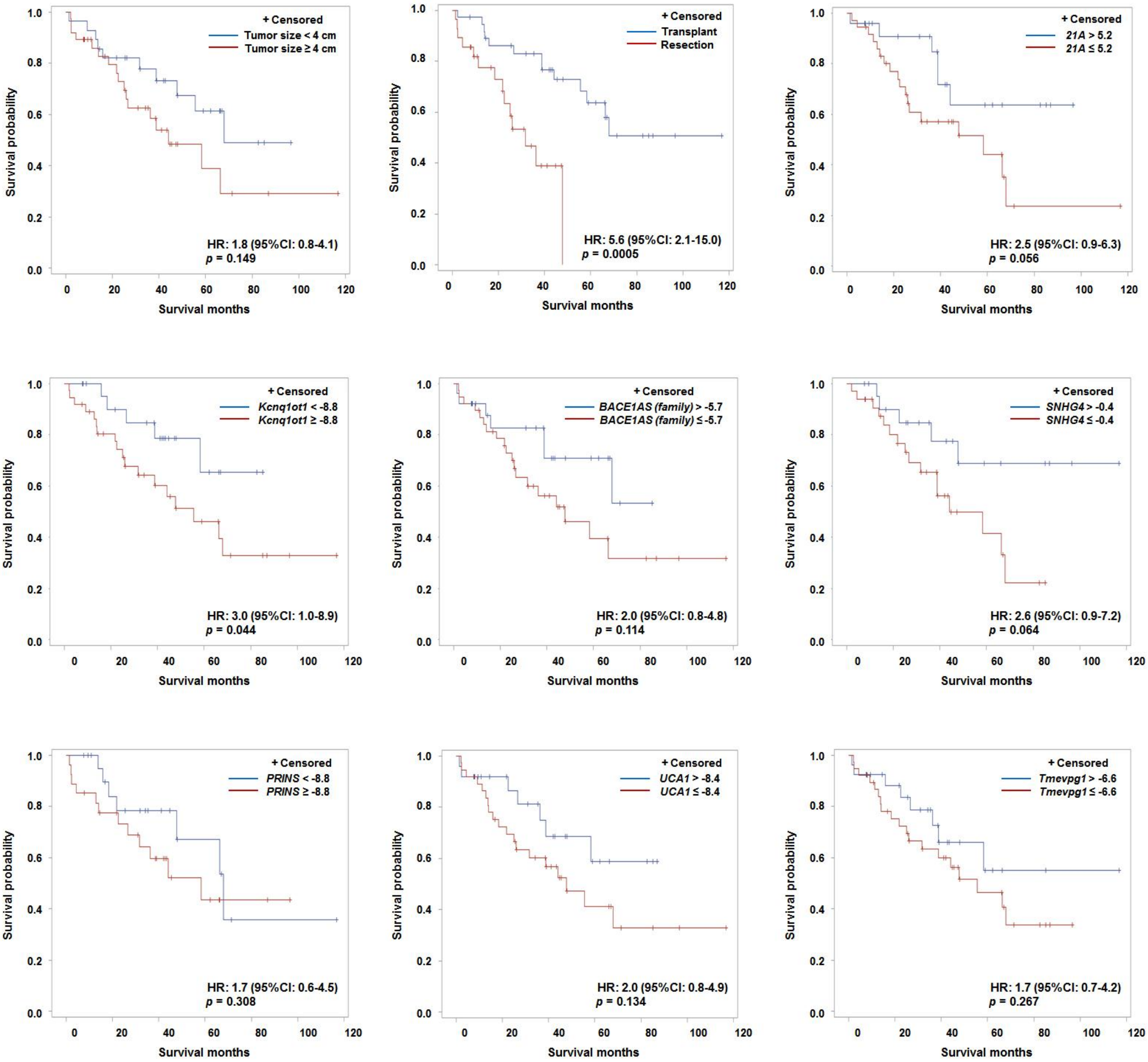

Figure S3. Expression of individual lncRNA (median expression level was used as the cutoff), tumor size (≥4 cm *vs.* <4 cm) and treatment (resection *vs.* transplant) in prediction of HCC survival time. Seven lncRNAs (*Kcnq1ot1*, *21A*, *SNHG4*, *BACE1AS* (family), *PRINS*, *UCA1*, *Tmevpg1*) can individually predict a shorter HCC survival time with hazard ratios (HR) ranging from 1.7 to 3.0 adjusted for age and gender, similar to that for large tumor size (HR = 1.8, 95%CI: 0.8–4.1) and resection treatment (HR = 5.6, 95%CI: 2.1–15.0).

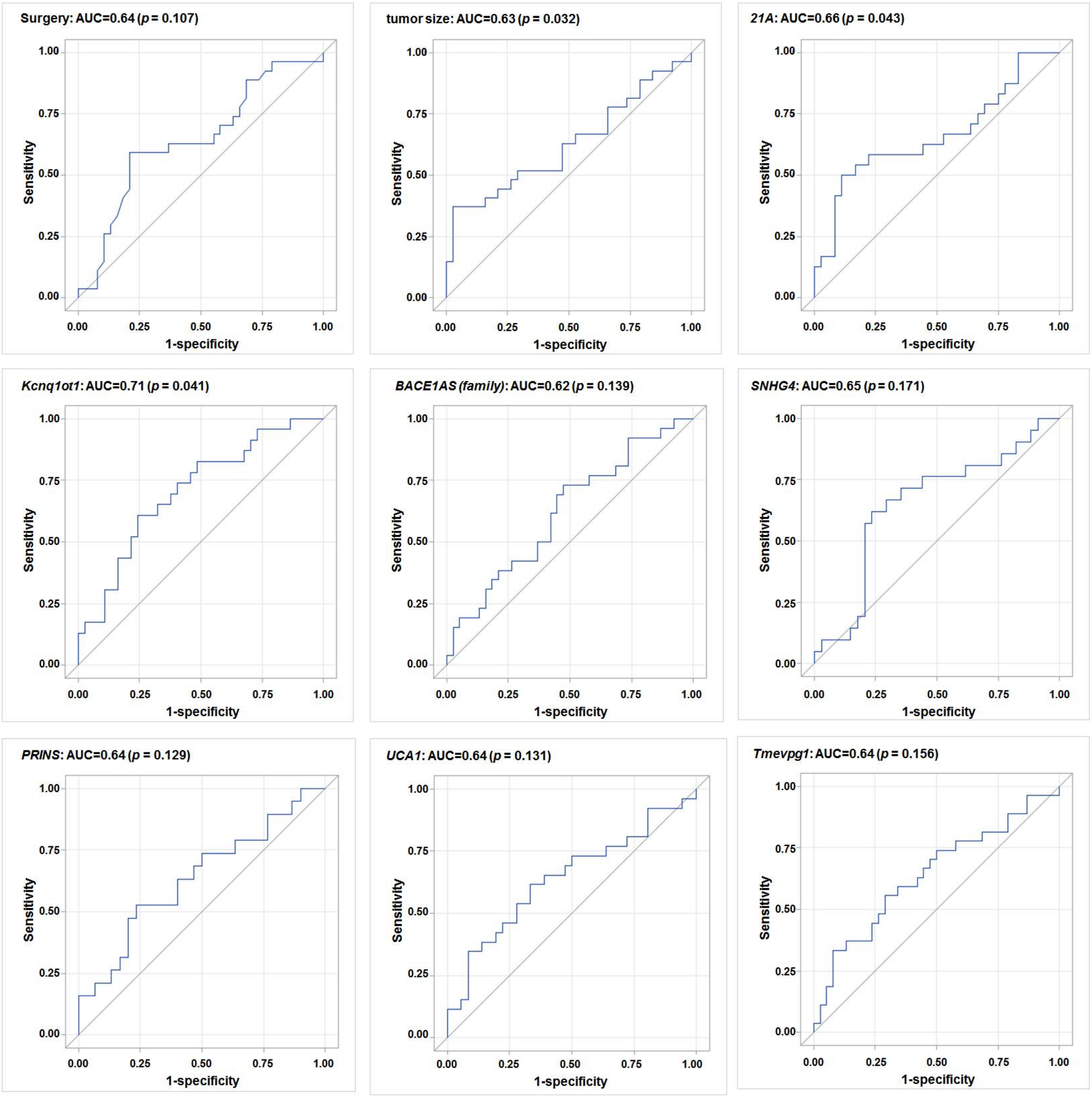

Figure S4. The ROC curves of individual lncRNAs, tumor size and treatment for HCC survival. The predictive accuracy for individual lncRNA ranges from 0.62 to 0.71, similar to the AUCs of large tumor size (0.63) and resection treatment (0.64) adjusted for age and gender.

Table S1. Name and location of lncRNAs detected in the Lnc Profiler™ qPCR Array.

	**1**	**2**	**3**	**4**	**5**	**6**	**7**	**8**	**9**	**10**	**11**	**12**
A	21A	7SK	7SL	Air	AK023948	Alpha 280	Alpha 250	ANRIL	anti-NOS2A	antiPeg11	BACE1AS	BC200
B	CAR Intergenic 10	DHFR upstream transcripts	Dio3os	DISC2	DLG2AS	E2F4 antisense	EgoA	EGO B	Emx2os	Evf1 and EVF2	GAS5-family	Gomafu
C	H19	H19 antisense	H19 upstream conserved 1 & 2	HAR1A	HAR1B	HOTAIR	HOTAIRM1	HOTTIP	Hoxa11as	HOXA3as	HOXA6as	HULC
D	IGF2AS	IPW	Jpx	Kcnq1ot1	KRASP1	L1PA16	p21	RoR	SFMBT2	VLDLR	LOC285194	LUST
E	Malat1	mascRNA	MEG3	MEG9	MER11C	ncR-uPAR	NDM29	NEAT1	Nespas	NRON	NTT	p53 mRNA
F	PCGEM1	PR antisense transcripts	PRINS	PSF inhibiting RNA	PTENP1	RNCR3	SAF	SCA8	snaR	SNHG1	SNHG3	SNHG4
G	SNHG5	SNHG6	Sox2ot	SRA	ST7OT	TEA ncRNAs	Tmevpg1	TncRNA	Tsix	TUG1	UCA1	UM9-5
H	WT1-AS	Xist	Y RNA-1	Zeb2NAT	Zfas1	Zfhx2as	18S rRNA	RNU43	GAPDH	LAMIN A/C	U6	No assay control

Table S2. Aberrant 7 lncRNAs in 65 HCC tumor tissues and prediction for HCC survival.

**lncRNAs**	**HCC Outcome**		**Fold-Change**	**Unadjusted *p*-value**
	**Dead**	**Alive**		
21A	2.1	4.4	−4.7	0.027
Kcnq1ot1	−6.8	−8.7	3.7	0.059
SNHG4	−1.7	−0.7	−2.0	0.103
BACE1AS (family)	−6.9	−5.6	−2.4	0.115
UCA1	−9.6	−8.6	−2.0	0.120
PRINS	−7.7	−9.0	2.5	0.131
Tmevpg1	−7.9	−6.9	−2.1	0.139
